# Resilience of Belgian Cattle Farmers Towards Infectious Diseases Outbreaks

**DOI:** 10.1155/tbed/2415909

**Published:** 2026-05-07

**Authors:** Véronique Renault, Laurent Delooz, Marie-France Humblet, Claude Saegerman

**Affiliations:** ^1^ Research Unit in Epidemiology, Risk Analysis and Biosecurity Applied, Fundamental and Applied Research for Animal Health (FARAH) Centre, Faculty of Veterinary Medicine, University of Liege, Liege, Belgium, ulg.ac.be; ^2^ Regional Association for Animal Health and Identification (ARSIA), Ciney, Belgium; ^3^ Department for Occupational Protection and Hygiene—Unit Biosafety, Biosecurity and Environmental Licences, University of Liege, Liege, Belgium, ulg.ac.be

**Keywords:** biosecurity, cattle, effectiveness, farmer, feasibility, implementation, infectious diseases, outbreaks, resilience, scoring

## Abstract

The study addresses the growing risk of infectious disease outbreaks in livestock by developing and applying a composite resilience index to evaluate the adaptive capacity of Belgian cattle farmers in implementing biosecurity measures (BSMs). The framework integrates three core dimensions, that is, implementation, feasibility and effectiveness, across three priority diseases representing distinct epidemiological profiles and transmission routes like brucellosis, bovine viral diarrhoea (BVD) and foot‐and‐mouth disease (FMD). The analysis combines three data sources as follows: (i) a farm‐level survey documenting the implementation of 41 BSM across 100 cattle farms, (ii) a feasibility assessment conducted with a panel of 38 farmers and (iii) expert evaluations of the disease‐specific effectiveness of each BSM. Farm‐level resilience scores were computed by weighting the degree of implementation by the feasibility and effectiveness of each BSM. Results show marked heterogeneity in resilience scores (17% to 99%) and in both the feasibility and adoption of BSM. Several measures assessed as both effective and feasible exhibited low uptake, indicating the existence of behavioural and perceptual barriers. The overall resilience index followed a normal distribution and was significantly higher in dairy farms than in beef farms, while no significant differences were observed between provinces. The study introduces a robust, evidence‐based tool for assessing and benchmarking farm‐level biosecurity resilience. It concludes that enhancing resilience depends less on the number of BSM implemented than on the strategic selection of context‐appropriate, feasible, and effective measures. The findings support a shift towards disease‐specific, tailored biosecurity strategies and highlight the need for further research into the behavioural determinants of feasibility.

## 1. Introduction

Resilience refers to the capacity of an individual or system to recover from crises or to absorb perturbations while maintaining its trajectory of development. ‘It is linked to dynamics of social systems encompassing adaptability, transformability, capacity of communities, and complex socio‐ecological systems to learn, cope, adjust and reorganise in response to shocks and stresses’ [1]. ‘It has also been defined as the capacity to sustain a given developmental pathway despite incremental and abrupt expected or unexpected changes and to adapt, refine and innovate within that pathway’ [2].

The concept of resilience has been widely mobilised across disciplines to analyse how individuals and systems respond to climatic, social, economic and political perturbations. In livestock systems, research over recent decades has focused extensively on the resilience of farmers in arid and semi‐arid regions, where production systems are exposed in response to acute climatic, social and political challenges [1, 3]. Yet, slower, more insidious forms of change have also reshaped livestock farming in other contexts. These include rising concerns about food security, the evolution of preventive veterinary medicine and the growing prominence of One Health approaches. The increasing frequency and geographic expansion of re‐emerging diseases have become a major threat to both the livestock sector and public health systems. Recent examples include recurrent Ebola virus outbreaks, the COVID‐19 pandemic, the spread of African swine fever (ASF) across Europe and Asia, outbreaks of monkeypox, and the northward expansion of multiple vector‐borne diseases within Europe [4–7].

Resilience, when understood as a complex and multi‐system concept, is strongly shaped by the capacity of individuals to cope with, adjust to and respond to evolving risks and disturbances. Several studies have examined the resilience of diverse farming systems in the face of climate change, economic crisis, and other major shocks over recent decades [1, 4]. However, the resilience of cattle farmers to large‐scale infectious disease outbreaks—specifically regarding their ability to prevent such events and mitigate their consequences—remains largely unexplored. The severe price crisis in the pork sector following recent ASF outbreaks in Europe and Asia illustrates the critical importance of addressing this gap, both for the farmers’ livelihood and for national and global economic stability [8, 9].

At the farm level, biosecurity measures (BSM) constitute essential tools for preventing the introduction and onwards transmission of infectious diseases. Numerous studies have assessed the degree to which such measures are implemented [10–16] and the factors shaping their adoption [17–21]. Most have reported low levels of BSM implementation among cattle farmers [10–13]. In Belgium, the most frequently cited reason for non‐adoption is the low perceived relevance or importance of BSM under routine conditions [11]. This perception is illustrated by statements such as ‘I do not have operational footbaths at the entrance to the barns, but I had them during the last foot‐and‐mouth (FMD) disease outbreak and would install them again in such situations.’ Strengthening and consolidating BSM on cattle farms, therefore, represents a critical yet insufficiently explored component of enhancing farmers’ resilience to infectious disease outbreaks, even though several studies have demonstrated associations between biosecurity levels, specific infectious diseases and/or productivity outcomes [22–26]. However, none of these studies assessed farmers’ actual capacity to adjust and upgrade their biosecurity practices in order to increase their resilience to major infectious disease threats.

The objective of this study is to construct a farmer‐level resilience score grounded in their capacity to enhance biosecurity practices, taking into account their current degree of implementation as well as the effectiveness and feasibility of each BSM. A further aim is to identify priority areas for improvement and to determine which BSM should be strategically adopted to strengthen the resilience of cattle farmers to infectious disease outbreaks (prioritisation of BSM).

## 2. Materials and Methods

The resilience—or individual adaptive capacity—of cattle farmers to strengthen and adjust BSM in order to better prevent or manage an infectious disease outbreak depends simultaneously on the current level of implementation, the effectiveness of BSM and its feasibility within the specific farm context. Evidence from Belgium [11] indicates that the principal determinants of whether a given BSM is implemented are its perceived importance (often linked to the expected impact of the disease on farm productivity), its efficiency and its practical feasibility. These determinants were therefore integrated into the construction of a composite resilience score designed to quantify each farmer’s capacity to adapt and respond to infectious disease threats. This approach acknowledges that when all relevant BSM are already fully implemented, further improvement is constrained unless the production system undergoes substantial structural change. Conversely, when resilience enhancement is possible, additional BSM should be selected and prioritised based on their effectiveness while ensuring that their feasibility aligns with the operational, economic and structural realities of the farm.

A previous Belgian study [11] assessed the level of implementation of BSM and examined farmers’ perceptions of its overall importance and efficiency, but it did not evaluate its actual feasibility in practice. To address this gap, an additional survey was conducted using the same list of BSM to gather farmers’ assessments of the practical feasibility of each BSM under real farm conditions. Moreover, an expert elicitation was organised to determine the disease‐specific effectiveness of each BSM efficiency for two reasons: (i) the initial survey captured only farmers’ perceptions of the general importance of BSM, without distinguishing between diseases and (ii) expert opinion grounded in scientific knowledge of pathogen biology and transmission dynamics provides a more accurate basis for assigning effectiveness scores to individual BSM.

### 2.1. Actual Level of Implementation of BSMs

The level of implementation of BSM by cattle farmers was assessed using data collected through face‐to‐face interviews with 100 cattle farmers [11]. The 47 BSMs evaluated in this survey were grouped into eight categories and then reclassified and consolidated into 41 BSMs targeting the prevention of disease introduction and/or transmission (Supporting information Table S1). For each BSM, implementation scores were standardised on a continuous scale from 0 (not implemented) to 1 (fully implemented), providing a comparable metric across farms and measures.

### 2.2. Feasibility of BSMs

Feasibility was evaluated using a structured questionnaire (Supporting Information, Table S2) in which cattle farmers rated the feasibility of each BSM on a scale from 0 (not feasible at all) to 5 (highly feasible), independently of its perceived importance or effectiveness. The list of BSM was derived from the previous Belgian study on implementation levels [11] to facilitate the consolidation of the datasets.

The survey was conducted during the first quarter of 2020 by the Regional Association for Animal Health and Identification (ARSIA) at three cattle farmer meetings. Participation was voluntary and free of charge. These meetings primarily aimed to disseminate up‐to‐date information on animal health, with particular emphasis on infectious bovine rhinotracheitis (IBR), bovine viral diarrhoea (BVD) and infectious causes of reproductive disorders.

When complementary BSM were merged into a single measure (e.g., testing animals to identify potential carriers and immediately removing them), the lowest feasibility score was retained to ensure a conservative assessment. Because feasibility scores did not follow a normal distribution, as confirmed by a Shapiro test, the median feasibility score for each BSM was used in the resilience calculations.

### 2.3. Weighting of BSM Categories and Assessment of Their Effectiveness

As these parameters depend on the epidemiological characteristics of infectious diseases, the assessment focused on three pathogens representing distinct disease typologies and transmission pathways. Bovine brucellosis (BRU) is a globally distributed zoonotic disease primarily caused by *Brucella abortus*. Although officially eradicated in Belgium, it remains a notifiable disease associated with substantial economic losses due to both direct and indirect impacts on animal productivity, including infertility, abortion, mastitis and reduced milk yield [26]. Transmission occurs through close contact with infected animals, exposure to uterine secretions or aborted foetuses, vertical and sexual transmission, and ingestion of contaminated feed or water [27–29]. BVD is endemic in Belgium and in most cattle‐producing countries, generating considerable financial losses mainly through its effects on reproductive performance and general health status [30, 31]. The principal route of introduction and spread is the presence of persistently infected calves, which acquire infection in utero during the first 4 months of gestation [32, 33]. Additional transmission pathways include animal‐derived products (milk, semen, embryos and slurry), people and fomites, aerosols and contaminated feed or water [30]. Wild ruminants such as roe deer are also susceptible to BVD infection [19] and may contribute—albeit to a limited extent—to viral introduction and dissemination. FMD is a highly contagious transboundary disease affecting domestic and wild cloven‐hoofed species. Although eradicated in Belgium, it remains a notifiable disease with major direct and indirect economic consequences, largely due to restrictions on the international trade of animals and animal products from affected regions, as well as the substantial costs associated with outbreak control [34]. The primary mode of transmission is direct contact with infected animals (including wildlife) or contaminated animal products [34]. Mechanical transmission via contaminated organic material, fomites, personnel or equipment moving between farms also plays a significant role [35]. In addition, airborne spread is possible, allowing the virus exhaled by infected animals to travel long distances depending on wind speed and direction [36, 37].

Three panels of 11–12 disease specialists were convened for BRU, BVD and FMD. Each panel provided assessments of the relative weight of each BSM category and the effectiveness of each individual BSM in preventing disease introduction and in limiting transmission both within and between herds.

For each disease, experts were asked to rank the importance of the BSM categories for (i) preventing pathogen introduction and (ii) reducing onwards transmission. They allocated a total of 100 points across the categories, assigning higher scores to categories considered more effective [38]. The number of points attributed to each category was then divided by the number of BSMs it contained, yielding a per‐measure weight for disease introduction (Weight_I) and transmission (Weight_T). This procedure was applied to all BSM except the measure ‘maintaining a closed herd’ within the ‘animal movements’ category. Because the adoption of a closed herd renders the other measures in this category irrelevant, this single BSM was assigned the full category weight.

Subsequently, within each BSM category, experts were asked to score the effectiveness of every measure in preventing disease introduction (Eff_I) and disease transmission (Eff_T) on a 0–100 scale, where 0 indicated ‘fully ineffective’ and 100 indicated ‘fully effective.’ These effectiveness scores were then normalised by dividing each value by 100, resulting in a continuous scale ranging from 0 to 1.

### 2.4. Resilience Score

**Figure 1:**
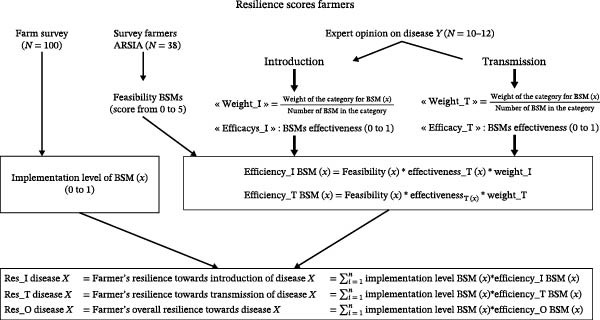
Methodological framework for estimating farmers’ disease‐specific resilience scores. Legend:  ^∗^ *N*: number of respondents to each of the surveys; *n* = number of biosecurity measures included in the survey; Res_I: resilience scores to disease introduction; Res‐T: resilience scores to disease transmission; Res_O: overall disease‐specific resilience score; BSM: biosecurity measures.

Three disease‐specific resilience scores were calculated for each cattle farmer as illustrated in Figure 1. For each pathogen (BRU, BVD and FMD), the analysis generated a score for resilience to disease introduction (Res_I_disease), to disease transmission (Res_T_disease) and an overall resilience score (Res_O_disease). All scores were expressed as a percentage of the maximum achievable value. Because the distributions of BSM feasibility and category weights were not expected to be normal, a pattern confirmed by statistical testing, median values were used in all subsequent calculations. In addition to the disease‐specific metrics, a global resilience score was computed for introduction (I), transmission (T) and overall resilience (O) according to the formulas presented as follows:

(1) Global resilience to disease introduction=Res_I_BRU+Res_I_BVD+Res_I_FMD,

(2) Global resilience to disease transmission=Res_T_BRU+Res_T_BVD+Res_T_FMD,

(3) Global resilience score to diseases=Res_O_BRU+Res_O_BVD+Res_O_FMDc.

Differences in the global resilience score between provinces and between farm types (beef vs. dairy) were evaluated using a Kruskal–Wallis test and a Wilcoxon test, respectively. Comparisons of disease‐specific resilience scores, as well as global resilience scores, were likewise performed using a Kruskal–Wallis test.

## 3. Results

### 3.1. Implementation and Feasibility of BSMs

The level of implementation of various BSM was assessed in a previous study [11].

To assess the feasibility of BSM, a field survey was launched in 2020 but had to be prematurely discontinued due to the COVID‐19 pandemic and the associated restrictions on planned stakeholder meetings. As a result, only three meetings could be held, yielding responses from 38 cattle farmers in southern Belgium (Wallonia).

Farmers rated the feasibility on a scale from 0 (not feasible at all) to 5 (highly feasible). Most BSM received scores spanning the full range. Two measures achieved a median feasibility score of 5: verifying the sanitary status of the source farm before purchasing animals and avoiding the sharing of reproductive animals between farms. In contrast, six measures obtained a median score of 1 or 0, indicating very low feasibility. These included installing double‐fencing around pastures, keeping barn doors closed, cleaning and disinfecting vehicles entering the premises, establishing a dedicated isolation area for sick animals, washing hands and changing boots or clothing when moving between barns, and ensuring that neighbouring farms do not spread manure within a 500‐m radius. Detailed results of the feasibility survey are provided in Supporting Information, Figure S1.

### 3.2. Category Weights and Effectiveness of BSMs

The panels evaluating weights and the effectiveness of BSM consisted of 11 experts for BRU and BVD and 12 experts for FMD. The resulting category weights are presented in Table 1. Across diseases, the highest weights for preventing pathogen introduction were consistently assigned to measures related to animal movements, animal health management and the prevention of direct contacts with shedders or carriers. For the prevention of disease transmission, experts attributed the greatest weight to BSM concerning animal health management and the prevention of contact with infected animals, followed by measures involving people and visitors.

**Table 1:** Median effectiveness weights for the different categories of biosecurity measures based on an expert elicitation conducted in 2020.

Categories of biosecurity measures	Median weight for disease introduction			Median weight for disease transmission		
	BRU	BVD	FMD	BRU	BVD	FMD
Cat. 1‐ Measures related to animal movements	28	45	29			
Cat. 2‐ Measures related to animal health management	18	18	10	20	20	15
Cat. 3‐ Measures related to the prevention of direct contacts with external shedders or carriers	16	20	15	30	35	25
Cat. 4‐ Measures related to people and visitors	8	5	12	10	8	14
Cat. 5‐ Measures related to vehicles and equipment	10	5	10	10	5	14
Cat. 6‐ Measures related to feed and water	5	1	6	10	3	8
Cat. 7‐ Measures related to animal products	5	2	8	5	4	5
Cat. 8‐ Measures related to general hygiene and management				10	13	12

Abbreviations: BRU, brucellosis; BVD, bovine viral diarrhoea; Cat., category; FMD, foot‐and‐mouth disease.

In a subsequent step, experts evaluated the effectiveness of each BSM in preventing either the introduction or the spread of the three diseases. Detailed results of this assessment are presented in Supporting Information, Figure S2, and Table 2 summarises all measures that achieved an effectiveness score of at least 80% in each scenario. For disease introduction, the most effective BSM—regardless of the pathogen—were those related to animal purchasing practices, specifically maintaining a closed herd and ensuring the safe origin of introduced animals. Quarantine measures received effectiveness scores below 80% for both BRU and FMD. For the prevention of disease transmission, BSM scoring ≥80% primarily targeted risks associated with direct contact with infected animals (identifying carriers, preventing contacts with animals from other farms and isolating sick animals) and indirect contact via fomites (visitors and equipment). For BRU specifically, additional highly effective measures (≥80%) included the presence of dedicated maternity pens and the proper management of foetal membranes and tissues following calving or abortion.

**Table 2:** Biosecurity measures scoring ≥80% effectiveness for disease introduction and disease transmission according to the 2020 expert elicitation.

Biosecurity measures		Effectiveness in preventing disease introduction			Effectiveness in preventing disease transmission		
		BRU	BVD	FMD	BRU	BVD	FMD
BSM 01	Maintaining a closed herd/no movement (re‐entries)	**X**	**X**	**X**			
BSM 02	Minimising purchasing and selling of animals		**X**				
BSM 04	Ensuring that purchased animals originate from a disease‐free source/no importation of infected animals	**X**	**X**	**X**			
BSM 05	Applying a 3‐week quarantine in a separate area or building for newly introduced animals		**X**				
BSM 07	Testing of re‐entering animals		**X**				
BSM 09	Continuous training on biosecurity	**X**			**X**		
BSM 13	Registering animal health data				**X**	**X**	
BSM 14	Identification and direct elimination of carriers or infected animals				**X**	**X**	
BSM 15	Dedicated isolation facility for sick animals				**X**		
BSM 16	No sharing of breeding animals with other farms	**X**		**X**	**X**		**X**
BSM 18	Avoid sharing or renting a pasture				**X**		**X**
BSM 19	Double fences (>2 m) around pastures				**X**		
BSM 20	Farmer/caretakers not working on, or visiting, other farms			**X**			**X**
BSM 21	Applying strict visitor access restrictions						**X**
BSM 28	Cleaning and disinfection of all contaminated equipment	**X**			**X**		
BSM 31	Safe origin of colostrum	**X**					
BSM 33	Safe origin of sperm	**X**					
BSM 35	Proper disposal of foetal membranes and tissues after calving or abortion				**X**		
BSM 39	Maternity pen separated from other areas				**X**		

Abbreviations: BRU, brucellosis; BSM, biosecurity measure; BVD, bovine viral diarrhoea; FMD, foot‐and‐mouth disease.

### 3.3. Resilience Score

**Figure 2:**
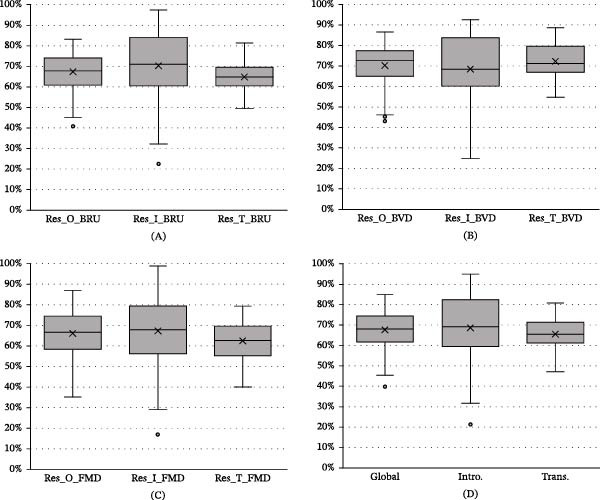
Global and disease‐specific resilience scores derived from the analysis.Legend: (A), brucellosis; (B), bovine viral diarrhoea; (C), foot‐and‐mouth disease; (D), global; BRU, brucellosis; BVD, bovine viral diarrhoea; FMD, foot‐and‐mouth disease; Intro., global score for disease introduction; Trans., global score for disease transmission.

Resilience scores—both disease‐specific and overall—varied across farms from 17% to 99% (Figure 2). The distribution of the global resilience score (calculated as the sum of the overall resilience scores for each disease) followed a normal distribution as confirmed by the Shapiro test.

When comparing the global resilience scores per province and farm type, no significant differences were detected between provinces. However, dairy farms exhibited significantly higher global resilience scores than those of other farm types. Additionally, the resilience scores for BRU and FMD transmission were significantly lower than the overall resilience and introduction scores (Table 3).

**Table 3:** Statistical outcomes (Kruskal–Wallis test) for the comparison of resilience scores in the 2020 Belgian study.

Disease	Variable	Coef.	Std. Err.	*p* >|*z*|	95% C.I.	
					Lower limit	Upper limit
BRU	Introduction	0.4792678	0.2635627	0.069	−0.0373057	0.9958413
	Transmission	−0.4838068	0.2339418	**0.039**	−0.9423243	−0.0252893
FMD	Introduction	0.21617	0.2554546	0.397	−0.2845119	0.7168519
	Transmission	−0.5148452	0.2362658	**0.029**	−0.9779176	−0.0517729
BVD	No significant differences					

*Note:* Bold values are used when *p*‐value is less than 0.05.

Abbreviations: BRU, brucellosis, BVD, bovine viral diarrhoea; FMD, foot‐and‐mouth disease.

## 4. Discussion

This study developed and applied an original resilience score to evaluate the adaptive capacity of cattle farmers in Belgium to enhance biosecurity in the context of infectious disease outbreaks. While several studies have examined farmers’ resilience to climatic or economic shocks, resilience in relation to infectious diseases has predominantly been assessed at the animal level, and recent work has highlighted the need to shift this perspective from the individual to the herd level [39]. By integrating three key dimensions of BSM—implementation, feasibility and effectiveness—this composite indicator provides a structured framework for assessing on‐farm resilience. The findings reveal substantial heterogeneity in farmers’ resilience levels and identify specific BSM that may serve as leverage points for targeted interventions.

### 4.1. Insights Into the Implementation and Feasibility of BSMs

The implementation data for each farm were extracted from a previous study [11] to assess the level of BSM implementation and to explore potential differences associated with farmer profiles or farm characteristics. The study reported an overall low level of BSM implementation and identified a positive correlation between the importance attributed by farmers to a given BSM and its actual implementation. Moreover, the persistent gap between knowledge and practice—often attributed to a low perceived risk, a high perceived burden or limited perceived effectiveness—remains a major constraint to improving biosecurity [11, 13, 17, 40–42].

This study shows that relatively simple yet effective measures—such as verifying the origin of purchased animals and testing them upon arrival, avoiding the sharing of reproductive stock, or retaining calves from feeding colostrum originating from other farms—were perceived as highly feasible and were widely implemented. In contrast, measures considered highly effective but reported as poorly feasible were rarely adopted. These include double‐fencing, strict vehicle disinfection procedures, a 3‐week quarantine for newly introduced animals and the establishment of a dedicated isolation area for sick animals. Together, these examples illustrate the persistent tension between epidemiological rigour and the practical constraints faced on farms. Several BSMs judged highly effective by the expert panel and highly feasible by farmers (score 4 or 5) were nonetheless implemented by fewer than 50% of the interviewed farmers. These BSM include minimising animal purchases and sales, installing footbaths at holding entrances, placing cadavers on cemented and covered areas, ensuring proper disposal of foetal membranes and tissues after calving or abortion, and applying hygienic measures for farm workers (farm‐specific boots and clothing and regular handwashing and disinfection). When examining the findings of the first study, in which farmers were asked to rate the importance of BSM, most measures considered unimportant by farmers were rated as important by experts. Hygienic measures for farm workers were an exception, being considered important by both experts and dairy farmers. This pattern is consistent with previous research [41] and underscores the complexity of behaviour determinants as multiple factors influence farmers’ decision‐making processes [41, 43]. It also highlights the farmers’ misperceptions regarding the importance of some BSM and the potential of targeted promotion strategies that prioritise BSM, offering an optimal balance between effectiveness and feasibility.

### 4.2. Effectiveness and Prioritisation of BSMs

Expert assessments highlighted the central importance of controlling animal movements and verifying the health status of incoming animals to prevent the introduction of diseases. This emphasis is consistent with the well‐documented role of the live animal trade in shaping disease dynamics and aligns with previous research [44–46]. For BVD and BRU, the introduction of infected animals or silent carriers is repeatedly identified as the primary pathway of pathogen entry into herds [46–49]. The consistently high weights attributed to these categories across the three diseases provide a strong rationale for prioritising them in surveillance strategies and communication efforts.

For disease transmission within farms, preventing direct contacts and controlling human‐mediated vectors—such as veterinarians, technicians, and visitors—emerged as critical components. This pattern is coherent with One Health principles, underscoring the need for integrative approaches that explicitly consider the animal–human environment interface [50].

Interestingly, quarantine—although widely promoted in disease‐control protocols—received comparatively low effectiveness ratings, particularly for BRU and FMD. This likely reflects the specific transmission dynamics of these pathogens, such as the potential for long‐distance airborne spread in the case of FMD. Nevertheless, previous studies and other expert panels have consistently identified quarantine as a highly important measure overall and as particularly relevant for BVD and FMD [41, 45]. This discrepancy underscores the need for context‐specific calibration of control guidelines, especially under field conditions where infrastructure, labour and enforcement capacity may be limited.

### 4.3. Implications of Resilience Score Patterns

The wide range of disease‐specific resilience scores (17%–99%) indicates substantial disparities in farmers’ adaptive capacity. While a minority of farms demonstrated near‐optimal ability to implement meaningful improvements, many remained highly vulnerable. The markedly lower transmission‐related resilience—particularly for FMD and BRU—further suggests that controlling secondary spread represents a critical weakness in the current system.

Not all measures contributed equally to resilience scores or to the ranking of the farm. The measure ‘keeping a closed herd’ exerted the strongest influence, reflecting both its high epidemiological value and the considerable variability in its feasibility across farms. This influence is consistent with the high weight assigned to this measure (28 on a 0–100 scale), in contrast with the much lower weights attributed to most other BSM (0.2–7). Conversely, several BSM—although epidemiologically relevant—had only minimal impact on score variation. These measures include:

• The application of a quarantine for re‐entering animals showed no measurable influence on resilience scores, although this result is likely biased by the fact that only 10% of the interviewed farmers were concerned by this measure.
• BSM related to arthropod and rodent control programmes also had limited influence, which can be explained by their low estimated effectiveness (median values between 0% and 3%, respectively). These measures may, however, play a more substantial role for other pathogens, particularly vector‐borne diseases [51].
• Two additional hygienic measures—those concerning the hygiene of professional visitors (clothing, boots and hand hygiene) and those related to compartment‐specific hygiene—also exerted minimal influence, largely because their implementation scores showed very limited variability across farms.

These findings indicate that broad, undifferentiated policies and recommendations are unlikely to be effective and should be complemented by disease‐specific, context‐adapted strategies that account for both the epidemiological relevance of each BSM and its practical feasibility on farms.

The integrated resilience score developed in this study is methodologically robust, combining expert‐derived weights with farmer‐reported feasibility and implementation data. Nonetheless, several limitations must be acknowledged. First, the feasibility assessment relied on a relatively small sample (*n* = 38), as COVID‐19 restrictions disrupted data collection, which may have limited the generalizability of feasibility estimates. Second, the weighting and effectiveness scoring were based on expert elicitation; although this approach is necessary in the absence of definitive field‐based effectiveness data, it may introduce subjective bias. Third, due to the COVID‐19 context, the feasibility study was conducted exclusively in Wallonia (southern part of Belgium), restricting the geographical representativeness of the results. Despite these limitations, the tool offers a valuable framework for benchmarking resilience and supporting strategic planning in veterinary public health and rural resilience. Repeating the feasibility survey with a larger and more diverse sample of famers would help determine whether farm‐type‐specific (e.g., dairy vs. beef) or region‐specific feasibility scores should be integrated, as these factors may significantly influence the outcomes.

## 5. Conclusion

This Belgian study introduces a novel composite resilience score designed to assess the capacity of cattle farmers to strengthen biosecurity practices in the face of infectious disease risks. The integrated approach demonstrates that improving resilience is not simply a matter of increasing the number of measures implemented but rather of prioritising those that are epidemiologically relevant, broadly applicable across farms, effective, feasible and variable enough in implementation to meaningfully differentiate resilience levels.

The findings highlight clear priority areas, notably the control of animal movements and the reduction of human‐mediated transmission pathways. Enhancing feasibility through targeted policy support—such as improved infrastructure, tailored training or economic incentives—could facilitate behavioural change and ultimately increase farmers’ resilience to infectious diseases.

Further research should investigate the dynamic use of the resilience score during active outbreaks and its potential integration into early warning or decision‐support systems. Understanding the behavioural determinants that shape farmers’ perception of feasibility also remains essential for designing effective interventions.

For policymakers and animal health authorities, this approach enables evidence‐based prioritisation of BSM and the identification of vulnerable farmer profiles, supporting more targeted and cost‐effective interventions. Within a broader One Health perspective, strengthening farmers’ resilience to infectious disease outbreaks contributes not only to agricultural sustainability but also to public health protection and ecosystem integrity.

## Funding

The study was supported by the University of Liege.

## Ethics Statement

This paper did not involve any experimental procedures, and all the data were anonymized. Ethical approval was, therefore, not required. The level of implementation of BSM by cattle farmers was assessed using data obtained from face‐to‐face interviews with 100 cattle farmers and presented in previously published information [11].

## Conflicts of Interest

The authors declare no conflicts of interest.

## Supporting Information

Additional supporting information can be found online in the Supporting Information section.

## Supporting information


**Supporting Information 1** Table S1. Biosecurity measures aimed at preventing the introduction and/or transmission of infectious diseases

**Supporting Information 2** Table S2. Feasibility survey of the 47 initial biosecurity measures in cattle farming

**Supporting Information 3** Figure S1. Results of the feasibility survey (*N* = 38)

**Supporting Information 4** Figure S2. Detailed survey results regarding the effectiveness of individual biosecurity measures

## Data Availability

The primary dataset used in this study is publicly available, and additional data may be obtained upon reasonable request from the corresponding author.
