# Supplementary material for: Resilience of Belgian Cattle Farmers Towards Infectious Diseases Outbreaks
Source: Transbound Emerg Dis. 2026 May 7;2026:2415909. doi: 10.1155/tbed/2415909 (PMC13150689; doi:10.1155/tbed/2415909)
Supplement: Supplementary file 1 — Supporting Information 1 Table S1. Biosecurity measures aimed at preventing the introduction and/or transmission of infectious diseases [file TBED-2026-2415909-s001.docx]

**Table S1. Biosecurity measures aimed at preventing the introduction and/or transmission of infectious diseases**

| **Cat** | **BSM** | **MEASURES PREVENTING THE INTRODUCTION AND/OR SPREAD OF INFECTIOUS DISEASE** | **SCORE** | **INTRODUCTION** | **TRANSMISSION** |
| --- | --- | --- | --- | --- | --- |
| **1** |  | **Related to animal movements** |  |  |  |
|  | 1 | Maintaining a closed herd / No movements ([re]entries) | 0: Animal purchases 4: No purchase but animals exiting and later returning to the premises  6: No movement | X |  |
|  | 2 | Minimizing purchases and sales of animals | 0: More than once per year 1: Once per year 2: Less than once per year | X |  |
|  | 3 | Applying an all in/all out system for each age group and each separate stable | 0: No 1: Yes | X |  |
|  | 4 | Ensuring a disease-free origin of animals / no importation of infected animals (checking health status, enquiring with veterinarian, etc.) | 0: No / Don't know 1: Sometimes 2: Yes | X |  |
|  | 5 | Maintaining a minimum 3-week quarantine period in a physically separate area or building for all newly introduced animals | 0: No quarantine period applied 1: Quarantine but not systematic and/or not in separated buildings and/or not for a min. of 15 days 2: Quarantine in a separated building for more than 15 days | X |  |
|  | 6 | Maintaining a minimum 3-week quarantine period in a physically separate area or building for animals returning to premises | 0: No / I don't know 1: Yes | X |  |
|  | 7 | Testing of animals before initial introduction or subsequent return | 0: No 1: Yes; but only purchased animals (not returning animals) 2: Yes, all | X |  |
|  | 8 | Ensuring optimal transport conditions, including safe handling, a clean vehicle, an adequate loading ramp, avoidance of overcrowding, calm handling of animals, minimal journey duration, and exclusion of routes involving a sorting centres | 0: No, potential contacts 1: No contacts | X |  |
| **2** |  | **Related to animal health management** |  |  |  |
|  | 9 | Maintaining up-to-date knowledge and competencies in biosecurity and animal health through continuous training | 0: No training 1: Training completed more than 1 year ago 2: Training completed less than 1 year ago | X | X |
|  | 10 | Implementing an adapted vaccination program (0= no vaccines or not in Belgium. or not relevant) | 0 to 6 | X |  |
|  | 11 | Implementing an arthropod control program | 0: No 1: Yes, for ectoparasites OR flying insects (repellent) 2: Yes, for both ectoparasites and flying insects | X | X |
|  | 12 | Implementing a rodent control program | 0: No 1: Yes, traps/rodenticides 2: Yes, a specialized firm intervening at least once per year | X | X |
|  | 13 | Maintaining an up-to-date animal identification and record-keeping register incorporating animal-health data | 0: No 1: Yes |  | X |
|  | 14 | Identification and elimination of carrier or infected animals through regular testing, including detection of persistently infected animals | 0: No direct elimination 1: Direct elimination |  | X |
|  | 15 | Placing sick animals in a designated quarantine facility | 0: No 1: Yes |  | X |
| **3** |  | **Related to the prevention of direct contacts with potential external shedders or carriers** |  |  |  |
|  | 16 | Avoiding the sharing of breeding animals with other farms | 0: Sharing 1: No sharing | X | X |
|  | 17 | Maintaining closed housing with locked doors to prevent contact with pets, carnivores, rodents, etc. in stables | 0: Access by all animals (dogs/cats/birds/rodents) 1: Dog access prohibited; access by cats, birds, and rodents still possible 2: No access permitted for dogs and cats; access by birds and rodents remains possible | X | X |
|  | 18 | Avoiding the sharing or renting of pastures | 0: sharing or renting of pastures 1: No sharing or renting of pastures (during the same season) | X | X |
|  | 19 | Preventing contact in pastures with animals from other farms and with wildlife (pigs and ruminants) using single or double fencing | 0: No 1: Yes, but contact with wildlife remains possible 2: Yes, no contact with other animals | X | X |
| **4** |  | **Related to people and visitors** |  |  |  |
|  | 20 | Preventing contact between the farmer or farm workers and animals originating from other farms | 0: Yes 1: No | X | X |
|  | 21 | Restricting visitor access | 0: No 1: Yes, with a few exceptions (1 to 2 categories) 2: Yes, except veterinarians and/or inseminators 3: Yes, for all professional visitors | X | X |
|  | 22 | Providing in-house or clean boots and clothing for visitors | 0: No 1: Yes | X | X |
|  | 23 | Enforcing appropriate personal hygiene practices for professional visitors, using their own equipment (boots, clothes, hand hygiene) | 0: No measures 1: Clean boots only, or clean boots and clothing but not for all professional visitors 2: Clean boots and clothing | X | X |
|  | 24 | Maintaining functional disinfection footbaths at the entrance of animal holdings | 0: No 1: Yes | X | X |
| **5** |  | **Related to vehicles/ equipment** |  |  |  |
|  | 25 | Restricting vehicle access, ensuring that(no vehicle enters areas where animals are kept and that any transit occurs via separated access roads | 0: Yes, access permitted for all vehicles  1: Preventing access exclusively for salesmen’s trucks 2: No vehicle access | X | X |
|  | 26 | Cleaning and disinfecting all incoming vehicles | 0: No or I don't know 1: Cleaning only 2: Cleaning and disinfection | X |  |
|  | 27 | Not sharing equipment or vehicles with other farms | 0: Yes 1: No | X | X |
|  | 28 | Cleaning and disinfecting all potentially contaminated equipment | 0: No 1: Yes | X | X |
| **6** |  | **Related to feed and water** |  |  |  |
|  | 29 | Storing animal feed in clean, enclosed structures to prevent contamination | 0: Accessible 1: No access for dogs and cats 2: No access for any animals at all | X | X |
|  | 30 | Preventing access to both running and stagnant water on pasture | 0: Yes  1: No | X | X |
|  | 31 | Purchasing external colostrum or calf milk exclusively from certified farms | 0: No, cow from another farm 1: Yes, cow from the same farm 2: Yes | X |  |
| **7** |  | **Related to animal products** |  |  |  |
|  | 32 | Preventing other farms from spreading manure within 500 m of the premises and retaining from using manure originating from external farms | 0: No 1:Yes | X |  |
|  | 33 | Ensuring the safe origin of semen and embryos | 0: No 1:Yes 9:No, artificial inseminations | X |  |
|  | 34 | Maintaining an appropriate carcass disposal system that prevents exposure to scavengers, including the use of a cemented area and carcass cover or sealed container) | 0: No 1: Cemented floor without cover 2: Cemented floor and cover |  | X |
|  | 35 | Immediate and proper disposal of foetal membranes and tissues following abortion and/or calving | 0: No specific disposal procedure 1: Disposed of in the slurry pit or on manure 2: Rendering services |  | X |
| **8** |  | **Related to general hygiene and management** |  |  |  |
|  | 36 | Housing density | 0: Not respected 1: Yes, respected for calves or for adults only 2: Yes, respected for both calves and adults |  | X |
|  | 37 | Removal of soiled bedding or litter and maintenance of fresh, clean bedding | 0: No 1: Yes, in some stables 2: Yes, for all stables and age groups |  | X |
|  | 38 | Personal hygiene practices of the worker/farmer (boots, clothing, hand hygiene, etc.) | 0: Nothing 1: 1 measure out of 3 2: 2 measures out of 3 3: All hygiene measures implemented |  | X |
|  | 39 | Having adapted, easy to clean, isolated and dedicated maternity pens | 0: Non-existing 1: Existing but direct contacts are possible 2: Existing, with no possibility of direct contact |  | X |
|  | 40 | Adults and young animals are housed in separate stables | 0: No 1: Yes, but no physical separation 2: Yes |  | X |
|  | 41 | Compartment-specific hygiene measures (hand cleaning and changing clothing/boots) | 0: No 1: Yes |  | X |

Legend: Cat, category; BSM, biosecurity measure.
