# Supplementary material for: Resilience of Belgian Cattle Farmers Towards Infectious Diseases Outbreaks
Source: Transbound Emerg Dis. 2026 May 7;2026:2415909. doi: 10.1155/tbed/2415909 (PMC13150689; doi:10.1155/tbed/2415909)
Supplement: Supplementary file 2 — Supporting Information 2 Table S2. Feasibility survey of the 47 initial biosecurity measures in cattle farming [file TBED-2026-2415909-s002.docx]

**Table S2. Survey on the feasibility of the initial 47 biosecurity measures in cattle farming**

| **Survey assessing the feasibility of 47 biosecurity measures in Belgian cattle farming (2020)** Your perspective as the person responsible for implementing these measures on a daily basis is highly valuable. Thank you for sharing it.  Please assign a feasibility score from 0 to 5 to each of the listed measures ( 0: “not feasible at all” to 5: " highly feasible") Please note that this score concerns only the practical or operational feasibility of the measure, and not its efficacy or benefits | | | | | | | |
| --- | --- | --- | --- | --- | --- | --- | --- |
|  |  |  |  |  |  |  |  |
| **N°** | **Biosecurity measure** | **In your view, what is the feasibility of this measure?  (0: not feasible at all; 5: highly feasible)** | | | | | |
| 1 | Maintaining a closed herd / No movements ([re]entries) | **🔾 0** | **🔾 1** | **🔾 2** | **🔾 3** | **🔾 4** | **🔾 5** |
| 2 | Minimizing purchases and sales of animals | **🔾 0** | **🔾 1** | **🔾 2** | **🔾 3** | **🔾 4** | **🔾 5** |
| 3 | Applying an all in/all out system for each age group and each separate stable | **🔾 0** | **🔾 1** | **🔾 2** | **🔾 3** | **🔾 4** | **🔾 5** |
| 4 | Ensuring a disease-free origin of animals / no importation of infected animals | **🔾 0** | **🔾 1** | **🔾 2** | **🔾 3** | **🔾 4** | **🔾 5** |
| 5 | Maintaining a minimum 3-week quarantine period in a physically separate area or building for all newly introduced animals | **🔾 0** | **🔾 1** | **🔾 2** | **🔾 3** | **🔾 4** | **🔾 5** |
| 6 | Maintaining a minimum 3-week quarantine period in a physically separate area or building for animals returning to premises | **🔾 0** | **🔾 1** | **🔾 2** | **🔾 3** | **🔾 4** | **🔾 5** |
| 7 | Testing of animals before initial introduction or subsequent return | **🔾 0** | **🔾 1** | **🔾 2** | **🔾 3** | **🔾 4** | **🔾 5** |
| 8 | Ensuring optimal transport conditions, including safe handling, a clean vehicle, an adequate loading ramp, avoidance of overcrowding, calm handling of animals, minimal journey duration, and exclusion of routes involving a sorting center | **🔾 0** | **🔾 1** | **🔾 2** | **🔾 3** | **🔾 4** | **🔾 5** |
| 9 | Implementing an adapted vaccination program (0= no vaccines or not in Belgium, or not relevant) | **🔾 0** | **🔾 1** | **🔾 2** | **🔾 3** | **🔾 4** | **🔾 5** |
| 10 | Avoiding the sharing of breeding animals with other farms | **🔾 0** | **🔾 1** | **🔾 2** | **🔾 3** | **🔾 4** | **🔾 5** |
| 11 | Maintaining an appropriate carcass disposal system that prevents exposure to scavengers, including the use of a cemented area and carcass cover or sealed container) | **🔾 0** | **🔾 1** | **🔾 2** | **🔾 3** | **🔾 4** | **🔾 5** |
| 12 | Use of double fencing to prevent contact with wildlife (pigs and ruminants) and with animals from other farms on pasture | **🔾 0** | **🔾 1** | **🔾 2** | **🔾 3** | **🔾 4** | **🔾 5** |
| 13 | Avoiding the sharing or renting of pastures | **🔾 0** | **🔾 1** | **🔾 2** | **🔾 3** | **🔾 4** | **🔾 5** |
| 14 | Maintaining closed housing with locked doors to prevent contact with pets, carnivores, rodents, etc. in stables | **🔾 0** | **🔾 1** | **🔾 2** | **🔾 3** | **🔾 4** | **🔾 5** |
| 15 | Implementing an arthropod control program | **🔾 0** | **🔾 1** | **🔾 2** | **🔾 3** | **🔾 4** | **🔾 5** |
| 16 | Implementing a rodent control program | **🔾 0** | **🔾 1** | **🔾 2** | **🔾 3** | **🔾 4** | **🔾 5** |
| 17 | Preventing contact between the farmer or farm workers and animals originating from other farms | **🔾 0** | **🔾 1** | **🔾 2** | **🔾 3** | **🔾 4** | **🔾 5** |
| 18 | Restricting visitor access | **🔾 0** | **🔾 1** | **🔾 2** | **🔾 3** | **🔾 4** | **🔾 5** |
| 19 | Providing in-house or clean boots and clothing for visitors | **🔾 0** | **🔾 1** | **🔾 2** | **🔾 3** | **🔾 4** | **🔾 5** |
| 20 | Enforcing appropriate personal hygiene practices for professional visitors, using their own equipment (boots, clothes, hand hygiene, etc.) | **🔾 0** | **🔾 1** | **🔾 2** | **🔾 3** | **🔾 4** | **🔾 5** |
| 21 | Maintaining functional disinfection footbaths at the entrance of animal holdings | **🔾 0** | **🔾 1** | **🔾 2** | **🔾 3** | **🔾 4** | **🔾 5** |
| 22 | Restricting vehicle access, ensuring that no vehicle enters areas where animals are kept and that any transit occurs via separated access roads | **🔾 0** | **🔾 1** | **🔾 2** | **🔾 3** | **🔾 4** | **🔾 5** |
| 23 | Cleaning and disinfecting all incoming vehicles | **🔾 0** | **🔾 1** | **🔾 2** | **🔾 3** | **🔾 4** | **🔾 5** |
| 24 | Ensuring that animal transport vehicles and other vehicles are leak-proof, cleaned, and disinfected prior to entry, and that they circulate through separate access routes. | **🔾 0** | **🔾 1** | **🔾 2** | **🔾 3** | **🔾 4** | **🔾 5** |
| 25 | Not sharing equipment or vehicles with other farms | **🔾 0** | **🔾 1** | **🔾 2** | **🔾 3** | **🔾 4** | **🔾 5** |
| 26 | Cleaning and disinfecting all potentially contaminated equipment | **🔾 0** | **🔾 1** | **🔾 2** | **🔾 3** | **🔾 4** | **🔾 5** |
| 27 | Storing animal feed in clean, enclosed structures to prevent contamination | **🔾 0** | **🔾 1** | **🔾 2** | **🔾 3** | **🔾 4** | **🔾 5** |
| 28 | Preventing access to both running and stagnant water on pasture | **🔾 0** | **🔾 1** | **🔾 2** | **🔾 3** | **🔾 4** | **🔾 5** |
| 29 | Purchasing external colostrum or calf milk exclusively from certified farms | **🔾 0** | **🔾 1** | **🔾 2** | **🔾 3** | **🔾 4** | **🔾 5** |
| 30 | Preventing other farms from spreading manure within 500 m of the premises | **🔾 0** | **🔾 1** | **🔾 2** | **🔾 3** | **🔾 4** | **🔾 5** |
| 31 | Ensuring the safe origin of semen and embryos | **🔾 0** | **🔾 1** | **🔾 2** | **🔾 3** | **🔾 4** | **🔾 5** |
| 32 | Pasteurization of raw milk and derived dairy products | **🔾 0** | **🔾 1** | **🔾 2** | **🔾 3** | **🔾 4** | **🔾 5** |
| 33 | Manure spreading only in the absence of wind | **🔾 0** | **🔾 1** | **🔾 2** | **🔾 3** | **🔾 4** | **🔾 5** |
| 34 | Maintaining an up-to-date animal identification and record-keeping register that includes animal health data | **🔾 0** | **🔾 1** | **🔾 2** | **🔾 3** | **🔾 4** | **🔾 5** |
| 35 | Continuous surveillance and monitoring (standardized disease definitions, scoring cards, rectal temperature measurements, record-keeping – including product, dose, route of administration, and withdrawal period – use of electronic systems, and integration of producer and veterinary records, etc. ) | **🔾 0** | **🔾 1** | **🔾 2** | **🔾 3** | **🔾 4** | **🔾 5** |
| 36 | Identification of carrier or infected animals through regular testing, including detection of persistently infected animals | **🔾 0** | **🔾 1** | **🔾 2** | **🔾 3** | **🔾 4** | **🔾 5** |
| 37 | Elimination of carrier and infected animals through regular testing, including detection of persistently infected animals | **🔾 0** | **🔾 1** | **🔾 2** | **🔾 3** | **🔾 4** | **🔾 5** |
| 38 | Allowing only healthy animals on common pastures (with prior testing) | **🔾 0** | **🔾 1** | **🔾 2** | **🔾 3** | **🔾 4** | **🔾 5** |
| 39 | Quarantine facilities for sick animals | **🔾 0** | **🔾 1** | **🔾 2** | **🔾 3** | **🔾 4** | **🔾 5** |
| 40 | Education on biosecurity and disease awareness | **🔾 0** | **🔾 1** | **🔾 2** | **🔾 3** | **🔾 4** | **🔾 5** |
| 41 | Housing density | **🔾 0** | **🔾 1** | **🔾 2** | **🔾 3** | **🔾 4** | **🔾 5** |
| 42 | Removal of soiled bedding or litter and maintenance of fresh, clean bedding | **🔾 0** | **🔾 1** | **🔾 2** | **🔾 3** | **🔾 4** | **🔾 5** |
| 43 | Personal hygiene practices of the worker/farmer (boots, clothing, hand hygiene, etc.) | **🔾 0** | **🔾 1** | **🔾 2** | **🔾 3** | **🔾 4** | **🔾 5** |
| 44 | Having adapted, easy to clean, isolated and dedicated maternity pens | **🔾 0** | **🔾 1** | **🔾 2** | **🔾 3** | **🔾 4** | **🔾 5** |
| 45 | Immediate and proper disposal of foetal membranes and tissues following abortion and/or calving | **🔾 0** | **🔾 1** | **🔾 2** | **🔾 3** | **🔾 4** | **🔾 5** |
| 46 | Adults and young animals are housed in separate stables | **🔾 0** | **🔾 1** | **🔾 2** | **🔾 3** | **🔾 4** | **🔾 5** |
| 47 | Compartment-specific hygiene measures (hand cleaning and changing clothing/boots) | **🔾 0** | **🔾 1** | **🔾 2** | **🔾 3** | **🔾 4** | **🔾 5** |
