# Supplementary figures and images for: Resilience of Belgian Cattle Farmers Towards Infectious Diseases Outbreaks
Source: Transbound Emerg Dis. 2026 May 7;2026:2415909. doi: 10.1155/tbed/2415909 (PMC13150689; doi:10.1155/tbed/2415909)

**Figure S1. Results of the feasibility survey (N=38)**

**
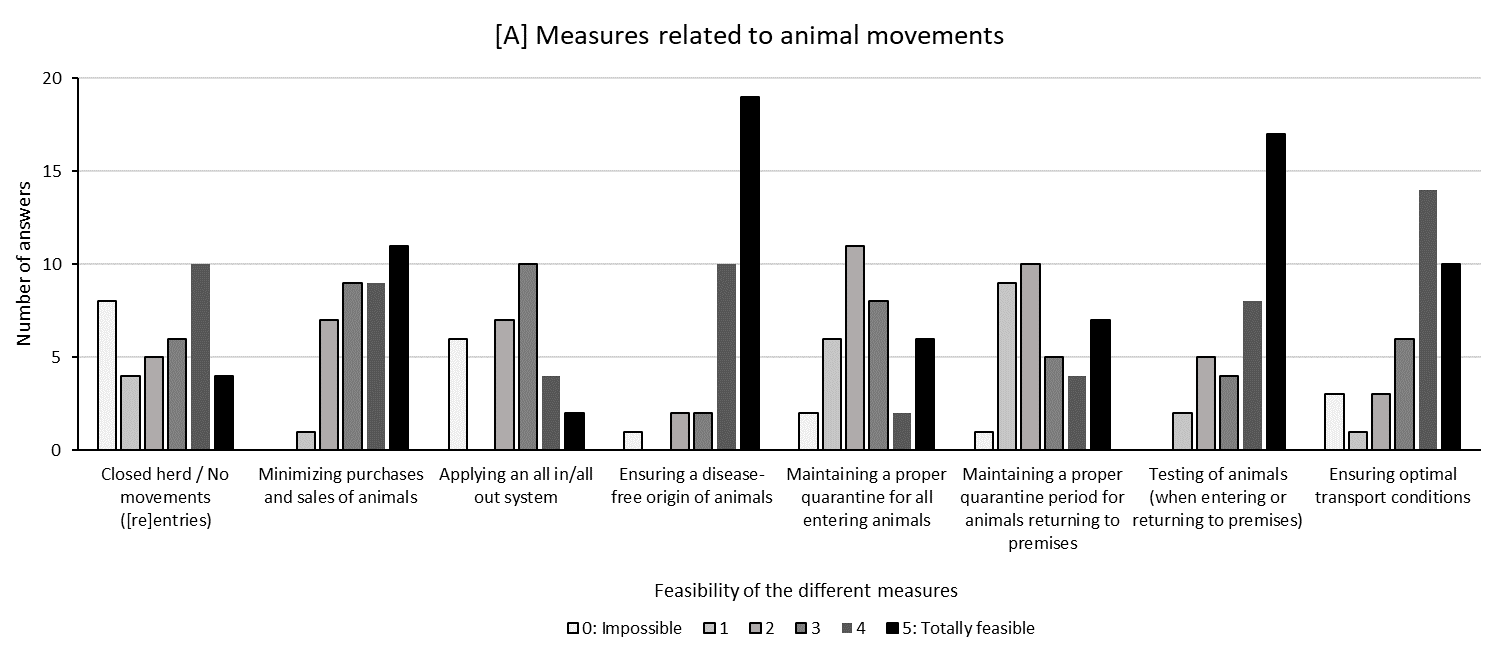
**

**
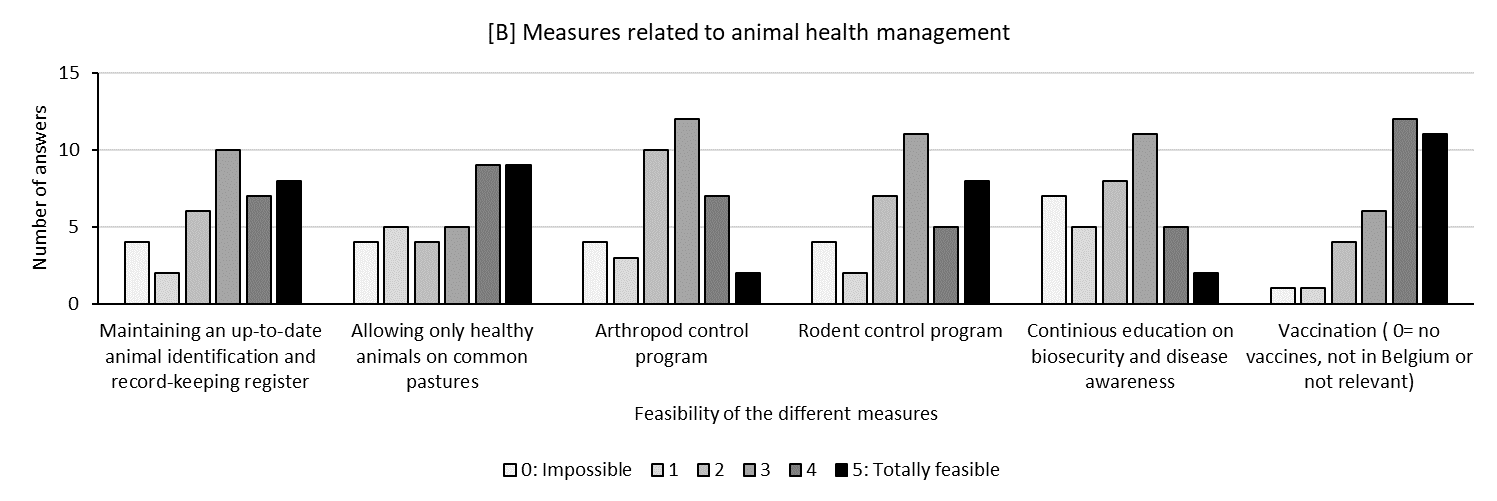
**

**
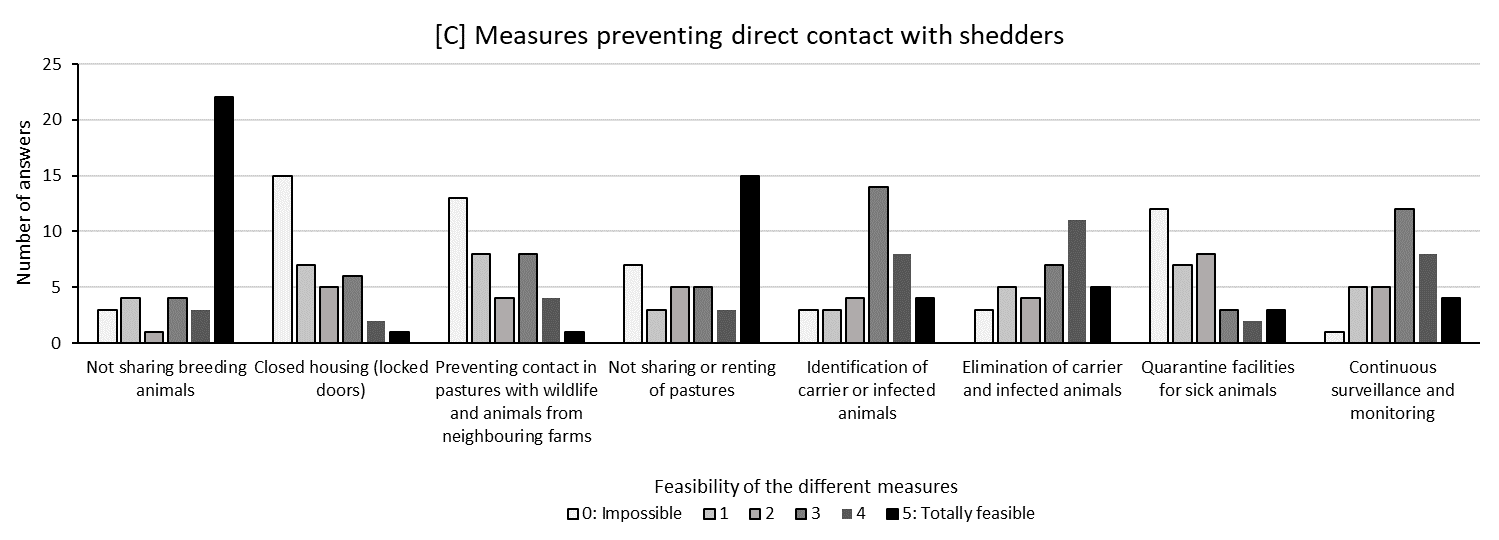
**

**
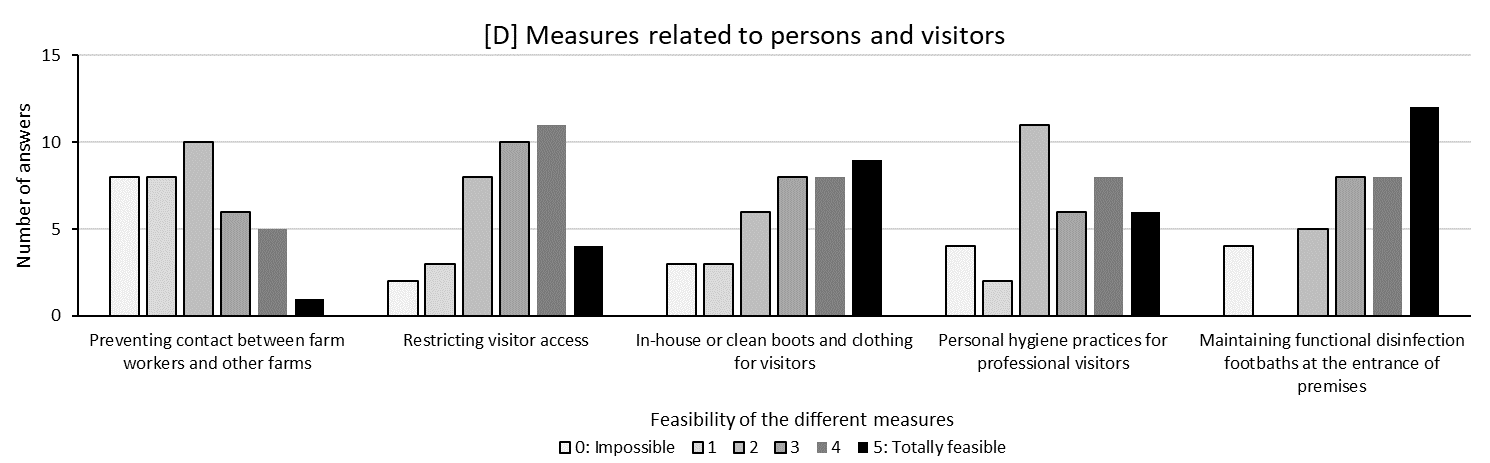
**

**
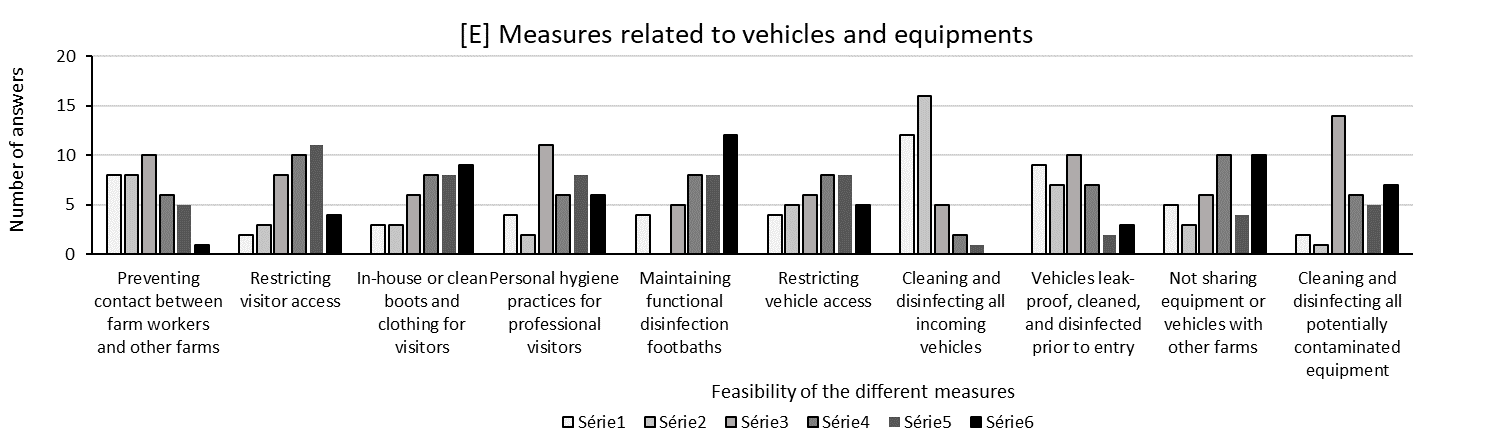
**

**
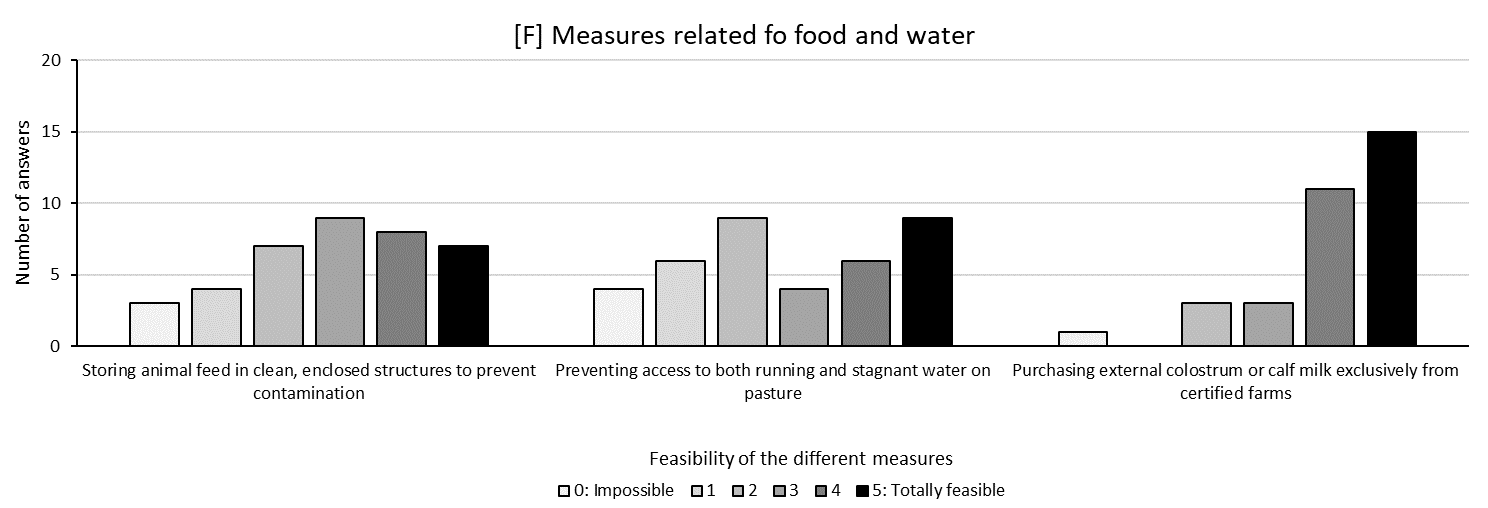
**

**
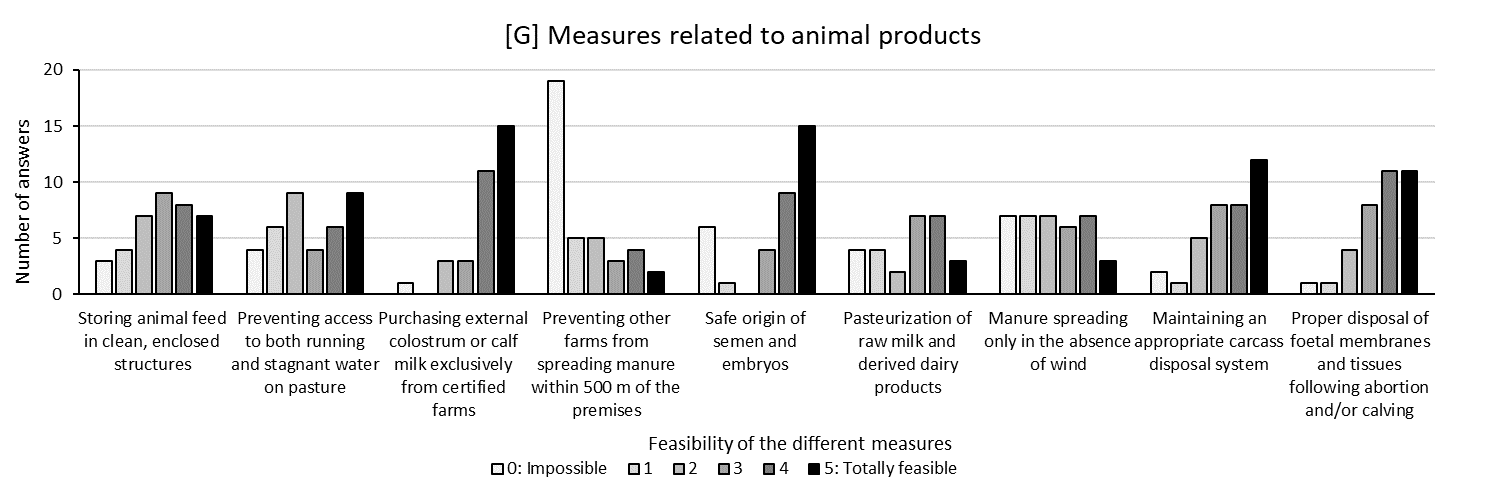
**


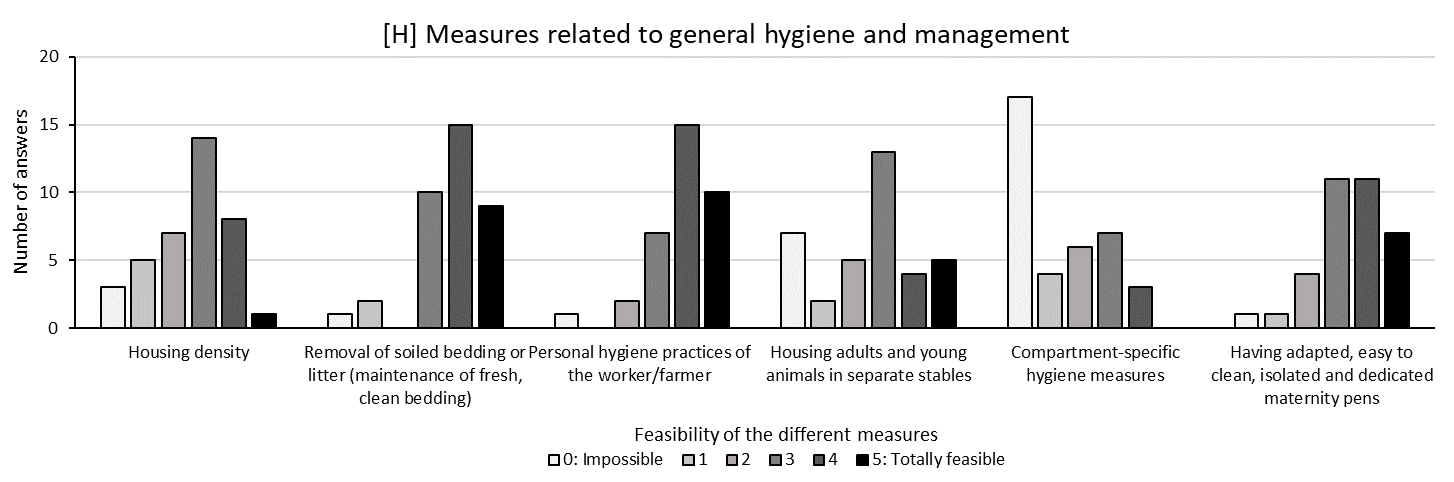

Supplement: Supplementary file 3 — Supporting Information 3 Figure S1. Results of the feasibility survey (N = 38) [file TBED-2026-2415909-s003.docx]
