# Supplementary material for: Resilience of Belgian Cattle Farmers Towards Infectious Diseases Outbreaks
Source: Transbound Emerg Dis. 2026 May 7;2026:2415909. doi: 10.1155/tbed/2415909 (PMC13150689; doi:10.1155/tbed/2415909)
Supplement: Supplementary file 4 — Supporting Information 4 Figure S2. Detailed survey results regarding the effectiveness of individual biosecurity measures [file TBED-2026-2415909-s004.docx]

**Figure S2. Sensitivity analysis of overall resilience scores and global resilience based on a Belgian survey (2020)**

[A] Overall resilience to brucellosis; [B] Overall resilience to bovine viral diarrhoea; [C] Overall resilience to foot and mouth disease; [D] Global resilience score; BSM 01 through BSM 41 are defined in Table S1.
